# An Untargeted Metabolomics Investigation of Jiulong Yak (*Bos grunniens*) Meat by ^1^H-NMR

**DOI:** 10.3390/foods9040481

**Published:** 2020-04-12

**Authors:** Chenglin Zhu, Massimiliano Petracci, Cheng Li, Enrico Fiore, Luca Laghi

**Affiliations:** 1 Department of Agro-Food Science and Technology, University of Bologna, 47521 Cesena, Italy; chenglin.zhu2@unibo.it (C.Z.); m.petracci@unibo.it (M.P.); 2 College of Food Science, Sichuan Agricultural University, Ya’an 625014, China; lichenglcp@163.com; 3 Department of Animal Medicine, Production and Health, University of Padova, 35100 Legnaro (Padova), Italy; enrico.fiore@unipd.it

**Keywords:** yak, meat, metabolome, proton nuclear magnetic resonance spectroscopy, pathway analysis

## Abstract

Yak represents the main meat source for Tibetan people. This work aimed to investigate the metabolome of raw meat from Jiulong yaks, focusing on specimens farmed and harvested locally through traditional procedures. Untargeted nuclear magnetic resonance spectroscopy (^1^H-NMR) was selected as the analytical platform. Samples from *longissimus thoracis*, *trapezius*, *triceps brachii* and *biceps femoris* muscles, with different prevalences of red and white fibers, were selected. Among the fifty-three metabolites quantified in each of them, carnitine, carnosine, creatine and taurine are known for their bioactive properties. Twelve molecules were found to be differently concentrated in relation to muscle type. *Longissimus thoracis*, compared to *biceps femoris*, had higher concentrations of carnosine and formate and lower concentrations of mannose, inosine, threonine, IMP, alanine, valine, isoleucine, tyrosine, phenylalanine and leucine. A metabolic pathway analysis suggested that the main pathways differing among the muscles were connected to the turnover of amino acids. These results contribute to a deeper understanding of yak raw meat metabolism and muscle type differences, which can be used as an initial reference for the meat industry to set up muscle-specific investigations. The possibility of simultaneously quantifying several bioactive compounds suggests that these investigations could revolve around meat’s nutritional value.

## 1. Introduction

Of the 14 million specimens of yak (*Bos grunniens*) bred in the world, 92% are located in the Himalayan highlands region [1,2], where they represent the main, if not the only, source of meat for people living in conditions made extreme by altitude. This is because its high erythrocyte count [3] and its efficient energy and nitrogen utilization [4] make yak well adapted to the paucity of oxygen and energy sources [5]. Its peculiar enzyme activity and gene expression profile [6] have positive consequences also on the meat quality characteristics, specifically on tenderness, juiciness and leanness [7]. These appreciable properties seem to be mainly linked to three muscles’ physiological features. Firstly, yak meat shows, throughout all the maturation steps, an intense protease activity [8] causing a pronounced myofibrillar denaturation and fragmentation in correspondence to the Z-line [9]. In addition, the final pH of yak meat is peculiarly distant from the isoelectric point [10]. Moreover, yak shows a lower intramuscular fat content in comparison with beef.

In the last decade, a new insight into the links between physiological properties of bovine meat and its quality characteristics has been offered by the advent of “omics” approaches. One example is offered by meta-proteomics, which was successfully applied to investigate the biomarkers of beef tenderness [11]. In this context, metabolomics seems particularly promising, because the cohort of meat’s low weight molecules, the so called metabolome, is considered as the most complete representation of its phenotype [12]. Indeed, up to now several aspects of meat quality have been explored from a metabolomics perspective [13], comprised of the effects of treatments such as aging or packaging conditions [14].

Among the platforms granting the needed high throughput, ^1^H-NMR spectroscopy has been widely used for investigations on cattle meat, because of its high reproducibility and the minimal sample preparation required. For example, Castejón et al. and Graham et al. [15,16] were able to evidence the metabolome profile evolution that occurs in beef tenderloins during its conservation. Ritota et al. [17] demonstrated the feasibility of employing meat’s metabolome to discriminate cattle breeds. Geographical origin [18,19] and meat’s treatments [20] were also studied through a metabolomics approach.

Despite the numerous applications of metabolomics in cattle meat, meat from the other bovine species has been rarely described. Examples can be traced only in the works by Ritota et al. [17], who observed buffaloes in relation to bovines, and in the work by Luo et al., who quantified 17 amino acids in the meat of domestic and semi-wild yaks [21]. Moreover, metabolomic investigations have generally observed meat from any bovine species as a matrix with homogenic characteristics, without considering the peculiarities of the different muscles. This also includes the most basic peculiarities, such as the prevalence of white and red fibers.

In the desire to set the basis for the development of metabolomics investigations of yak’s meat, with the present paper we would like to outline its metabolome, as can be observed by ^1^H-NMR. For this purpose we focus on one of its sub-species, Jiulong, representing approximately 30% of all yaks [22] bred at present in China. Meat metabolome is the result of the genetics of the animal, together with the breeding conditions and with the slaughtering procedures. This is particularly true for yak, for which tradition can still play a key role in breeding and processing procedures. To obtain a realistic picture of Jiulong yak meat, we collected samples from a facility still conducted traditionally, located in the area where these animals are mainly bred. To give a comprehensive metabolomic picture of Jiulong yak’s meat, we consider four muscles known to show a marked difference in the ratio between red and white fibers.

## 2. Materials and Methods

### 2.1. Sampling

We collected raw meat samples from male Jiulong yaks (36 ± 2 months of age) in a public abattoir (without modern slaughtering equipment) located in the pastoral area of Litang County (altitude 4000 m), Sichuan, China, at the end of August. Yaks are generally slaughtered at this time of the year at three years of age, because they reach the highest weight and have optimal health conditions.

The yaks had been transported to the abattoir and held in lairage for 24 h with water supplementation until 3 h before slaughtering. The yaks had been sacrificed following the local traditional manual procedures [22]. As described by Graham et al. [16], forty-five minutes *post-mortem*, one side of each carcass had been hung by the Achilles tendon and the other side had been hung using the pelvic suspension.

Two hours later, from 10 consecutively slaughtered animals we collected 7 samples of *triceps brachii* (TB), 5 samples of *biceps femoris* (BF), 5 samples of *longissimus thoracis* (LT) and 3 samples of *trapezius* (TP). The samples, weighing approximately 10 g, were immediately placed in bags at 4 °C. The discrepancies in the number of samples are linked to practical reasons during the slaughtering procedures. In detail, the TP muscle was generally removed together with the head, so that only three samples were collected. In the case of the other muscles, some samples could not be collected due to the impossibility to slow down the slaughtering procedures. This resulted in a total of 20 samples for metabolomics investigation. We transported all samples in dry ice and stored them prior to analysis at −80 °C.

### 2.2. Metabolome Analysis

To create yak meat samples suitable for ^1^H-NMR analysis, we added two grams of meat to 12 mL of distilled water, and then we homogenized the mixture for 5 min by means of a high-speed disperser (IKA, Staufen im Breisgau, Germany). Then, we centrifuged 1 mL of the mixed sample at 18,630 g and 4 °C for 15 min. To get rid of the fat fraction, we transferred 0.7 mL of the supernatant fraction to a new Eppendorf tube containing 0.8 mL chloroform, we vortex mixed for 3 min and we centrifuged again at the above conditions. We added the supernatants (0.5 mL) to 0.2 mL of a D_2_O solution, buffered at pH 7.00 ± 0.02 with a 1 M phosphate buffer, containing 3-(trimethylsilyl)-propionic-2,2,3,3-d_4_ acid sodium salt (TSP) 10 mM, used as NMR chemical-shift reference, and NaN_3_ 2 mM, used to avoid microbial proliferation. We finally centrifuged each sample at the above conditions.

We acquired ^1^H-NMR spectra at 298 K by means of an AVANCE III spectrometer (Bruker, Milan, Italy) operating at a frequency of 600.13 MHz. According to Zhu et al. [23], we suppressed the signals from broad resonances originating from large molecules with a CPMG-filter composed by 400 echoes with a 180° pulse of 24 μs and a τ of 400 μs, for a total filter of 330 ms. We suppressed the HOD residual signal through presaturation. We achieved this goal by employing the cpmgpr1d sequence, part of the standard pulse sequence library. We acquired each spectrum by summing up 256 transients, using 32 K data points over a 7184 Hz spectral window, with an acquisition time of 2.28 s. We adjusted the spectra baseline by detecting each peak according to the “rolling ball” strategy [24], implemented in the baseline R package [25]. We considered the differences in water content among samples by probabilistic quotient normalization (PQN) [26], applied to the entire array of spectra. We performed signals’ assignment by comparing their chemical shift and multiplicity with the Chenomx software library (Chenomx Inc., Edmonton, Canada, ver. 8.3), as shown in Figures S1–S12. We performed molecules’ quantification by taking advantage of TSP as a reliable internal standard. We integrated the signals for each molecule by means of rectangular integration. A graphic representation of the workflow for meat samples preparation and ^1^H-NMR spectra processing is shown in Figure S13.

### 2.3. Statistical Analysis

For statistical analysis we employed R computational language (ver. 3.6.1.) [27]. We transformed variables that were not-normally distributed according to Box and Cox [28]. Following Abadie et al. [29], we looked for molecules, whose concentration varied among muscle types, with ANOVA, followed by Tukey HSD post hoc test (*p* < 0.05). Due to the low number of samples in TP group, we investigated the differences between LT and TP groups focusing on fold change, as suggested by Wang et al. [30].

In order to obtain an overall of the trends underlying the metabolome of the samples, we calculated robust principal component analysis (rPCA) models [31] for the molecules accepted by the above described univariate analysis. For each rPCA model, we calculated the scoreplot, the projection of the samples in the PC space, tailored to highlight the structure of the data. In addition, we calculated the Pearson correlation to find out the relations between the concentration of each molecule and the components of the model.

We highlighted the most relevant metabolic pathways differing among the muscle types by pathway analysis, performed by means of MetaboAnalyst 4.0 [32]. For this purpose, we considered only the molecules whose concentrations were significantly different in the univariate analysis.

## 3. Results

### 3.1. ^1^H-NMR Spectra of Yak Raw Meat

**Figure 1:**
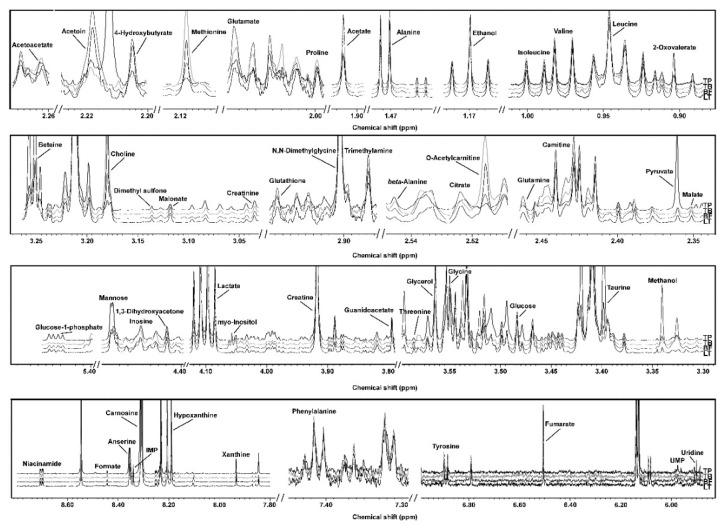
^1^H-NMR spectra from *trapezius* (TP), *triceps brachii* (TB), *biceps femoris* (BF) and *longissimus thoracis* (LT), representative of all the registered spectra. The name of each molecule appears over the signal used for its quantification. To ease the reader’s visual inspection, for each portion a spectrum with a convenient signal-to-noise ratio has been selected. The entire spectra and molecules identification is shown in Figures S2–S13.

The ^1^H-NMR spectra of each yak muscle type are reported in Figure 1, while molecules’ concentrations are reported in Table S1. A total of 53 molecules were identified and quantified, pertaining mainly to the chemical groups of amino acids, peptides and analogues, carbohydrates and derivates, organic acids and derivates, nucleosides, nucleotides and analogues. Among all the molecules characterized and quantified by ^1^H-NMR, carnitine, carnosine, taurine and creatine, are known as bioactive compounds. A bioactive compound is an extra nutritional constituent of food that can have a positive impact on body functions and may ultimately promote health [33].

### 3.2. Comparison among TB, BF and LT Muscles

Twelve of the quantified molecules were significantly different among TB, BF and LT muscles, namely carnosine, formate, mannose, inosine, threonine, IMP, alanine, valine, isoleucine, tyrosine, phenylalanine and leucine (Table 1).

**Figure 2:**
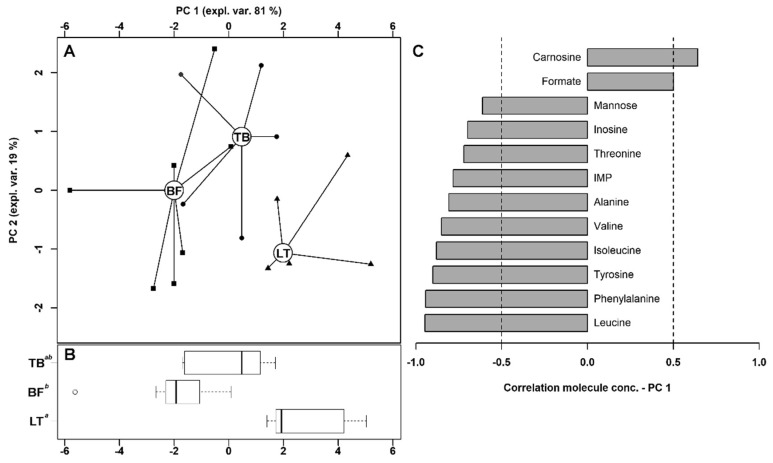
rPCA model built of these concentration of the molecules showing statistically significant differences among different groups. In the scoreplot (**A**), samples from the three groups are represented with triangles (LT), circles (TB) and squares (BF). The wide, empty circles represent the median of each samples’ group. In the boxplot (**B**), the position of the samples along PC 1 is summarized. Different superscript letters identify significantly different groups (*p* < 0.05). The loading plot (**C**) reports the correlation between the concentration of each substance and its importance over PC 1.

An rPCA model was built of these concentrations to obtain an overview of their trends, as shown in Figure 2.

The PC 1 of its scoreplot (Figure 2A), representing as much as the 81% of the entire samples’ variability so described, summarizes the differences between the samples collected from different muscles. In detail, LT muscle was found to be characterized by lower concentrations of mannose, inosine, threonine, IMP, alanine, valine, isoleucine, tyrosine, phenylalanine and leucine and higher concentrations of carnosine and formate.

The 12 molecules were used as a basis for a pathway enrichment analysis, to identify the most relevant pathways differentiating the muscles. Three pathways were highlighted: namely the valine, leucine and isoleucine biosynthesis pathway; the phenylalanine, tyrosine and tryptophan biosynthesis pathway and phenylalanine metabolism pathway.

### 3.3. Comparison between LT and TP Muscles

The last column of Table S1 lists the fold change differences of TP in comparison to LT samples. The concentrations of taurine and acetoin were considerably higher in LT samples, while the concentrations of glutamine, methionine, hypoxanthine, IMP, inosine, myo-inositol, choline, methanol and o-acetylcarnitine showed an opposite trend. In particular, the concentration of myo-inositol was 10 times higher in TP compared to LT samples.

**Figure 3:**
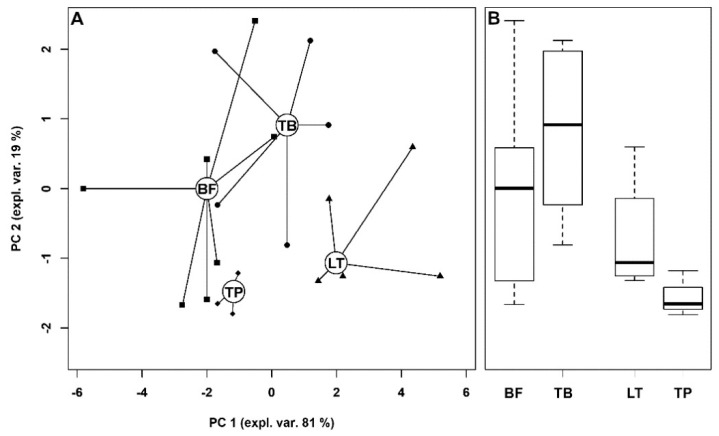
(**A**) Scoreplot of the rPCA model described in Figure 2, where TP samples have been projected. The wide, empty circles represent the median of the samples from the various types of muscles. (**B**) Boxplots summarizing the samples’ positions along PC 2.

To relate the metabolome of TP muscle with those of the other muscles, we treated the TP samples as an independent test set by projecting them over the rPCA described in Figure 2, as described by Figure 3. This choice evidenced that PC 2 was mainly influenced by muscle location along the yak, with the samples obtained from the legs characterized by positive PC 2 scores and the samples obtained from the *longissimus thoracis* and *trapezius* characterized by negative PC 2 scores. PC 1 scores appeared mainly as associated to the ratio between red and white fibers, with muscles with a prevalence of white fibers characterized by positive scores.

## 4. Discussion

This is the first study that characterizes, through an untargeted approach by ^1^H-NMR, raw yak meat metabolome. A total of 53 metabolites were unambiguously identified, a twofold number compared to previous studies on cattle beef investigated relying on the same analytical platform [13,16]. As a primer in the field, we devoted an intense effort to set up each aspect of the procedures for sample preparation and data mining so as to ease their reproduction. One of the main problems hindering ^1^H-NMR data reproduction is signals’ misalignment, caused by pH or ionic strength differences from sample to sample [12]. Figure 1 allows one to visually appreciate that the experimental conditions selected led to ^1^H-NMR spectra conveniently aligned, not requiring the application of algorithms for signal-by-signals shifting, differently from previous works [34]. In addition, Figures S2–S13 assist the reader in the signals’ assignment and quantification step, thus allowing the reproduction and double check of our assignments. These details are in line with the recommendations outlined by the EU Initiative “coordination of standards in metabolomics” (COSMOS) [35].

LT muscle is characterized by a peculiarly high number of white, fast-twitching glycolytic fibers. However, TP muscle harbors a higher number of slow-twitching oxidative fibers, while TB and BF can be considered as intermediate between the two [36,37,38]. Carnosine content is known to reflect the ratio between glycolytic and oxidative fibers. This molecule is therefore higher in white muscles, where it grants a high buffering capacity, thus compensating for the lactic acid accumulation connected to the anaerobic metabolism [39]. The concentration of this histidine-containing dipeptide we measured was coherent with such findings, being in LT muscle 42% higher than TB and 24% higher than BF. The comparison of the trends between the samples from TP and LT muscle seems a further confirmation, with the former characterized by higher concentration than the latter, in agreement with the observations in beef by Aristoy et al. [40]. From this point of view, some discrepancies in the literature must be evidenced. Mora et al. [41], for example, found similar carnosine concentrations in pigs in LT and BF, likely in connection with peculiarities connected to species and environment. Environment is expected to play a key role in the physiological differences between yak and other species or cattle breeds, with yak perfectly adapted to graze on steep slopes and in harsh environments.

Univariate analysis showed that the muscle type had a profound effect on the amino acids overall profile, with significant effects on the concentration of 7 amino acids of the 14 measured. Moreover, enrichment analysis showed that all the most altered pathways were related to amino acids metabolism. A similarly profound effect of muscle type on amino acids profile has also been observed for cattle [42], with consequences on important characteristics for meat quality like oxidative stability.

The fact that each of the seven amino acids showed a negative correlation with PC 1 scores suggests that the main reason for such trend is linked to the use of the muscles, more intense for legs and neck. The concentration of each of them was statistically different between BF and LT. Generally, TB showed intermediate values. An exception was represented by alanine, differently concentrated between TB and LT. Alanine is a non-essential amino acid, highly concentrated in muscles, where it serves as one of the major energy sources [43]. Goldstein et al. [44] noted that the release of alanine by muscle is a metabolic consequence of both the initial transamination reaction and its conversion into pyruvate via the normal oxidation pathway for the amino acids. In vivo, alanine can be produced from the breakdown of carnosine [45], which could explain the inverse relationship between the concentration of alanine and carnosine we noticed in the present investigation.

IMP pertains to the molecules derived from purines, often produced by ATP hydrolysis during the aging process of beef samples [46]. Dannert et al. [47] found that the concentration of IMP is not significantly different among the LT, BF and semimembranosus muscles of pork carcasses sampled 48 h post-mortem. However, we found in yak that the concentration of IMP was significantly higher in BF than in LT. This discrepancy suggests that the degradation phenomena occurring during muscles transformation into meat are likely to be, in yak, strongly muscle specific [48].

In spite of potential applications of the information obtained in the present work as a base for the valorization of yak’s meat, it is important to notice that among all the molecules characterized, four, namely carnitine, carnosine, taurine and creatine, are appreciated for their bioactive properties.

Carnitine is not an essential nutrient for humans, but 75% of it needs to be taken from food, mainly from meat. The major function of carnitine is to transport long chain fatty acids across inner mitochondrial membranes where the fatty acids are converted into biological energy by alpha-oxidation [49]. Similarly to carnitine, taurine is also a conditionally essential compound, because it cannot be synthesized in sufficient quantities by the human body. This substance has multiple functions and plays a significant role in many physiological processes, such as osmoregulation, immunomodulation and bile salt formation [50]. Creatine plays an important role in energy provision in muscle contraction, and its supplementation can increase the performance of muscles [51].

## 5. Conclusions

This is the first work devoted to the characterization of the metabolome of raw yak meat by means of untargeted ^1^H-NMR. As a primer in this context, we desired to analyze samples from the region where these animals are mainly bred and harvested through traditional procedures. This choice limited the number of samples analyzed, but a number of metabolites greater than previously reported for beef were still characterized. The metabolome profile which emerged appeared as strongly sensitive to the differences among muscles. This was particularly true for amino acids and degradation related molecules, suggesting therefore that the protocol described here could serve as a base for muscle specific studies of the transformations occurring in meat upon aging.

The present experiment showed that several bioactive molecules could be simultaneously and readily quantified by ^1^H-NMR, despite their chemical heterogeneity. The reference values listed in the present work set the basis for observations about the effects of processing practices on the nutritional value of meat, or for works aiming to valorize meat from this remarkable animal.

## Figures and Tables

**Table 1:** Molecules in yak raw meat with a concentration (mmol/g, median (IQR)) significantly different among muscle types.

	*Triceps brachii* (TB)	*Biceps femoris* (BF)	*Longissimus thoracis* (LT)	*p*
Alanine	4.76 × 10^−3^ (1.33 × 10^−3^) *^a 1^*	5.31 × 10^−3^ (4.77 × 10^−4^) *^a^*	3.14 × 10^−3^ (9.50 × 10^−4^) *^b^*	0.003
Carnosine	1.23 × 10^−2^ (1.65 × 10^−3^) *^b^*	1.60 × 10^−2^ (8.17 × 10^−3^) *^b^*	2.09 × 10^−2^ (1.84 × 10^−3^) *^a^*	0.017
Isoleucine	1.81 × 10^−4^ (3.39 × 10^−5^) *^ab^*	2.33 × 10^−4^ (6.07 × 10^−5^) *^a^*	1.69 × 10^−4^ (4.61 × 10^−5^) *^b^*	0.012
Leucine	3.54 × 10^−4^ (7.22 × 10^−5^) *^ab^*	4.70 × 10^−4^ (9.67 × 10^−5^) *^a^*	3.06 × 10^−4^ (9.42 × 10^−5^) *^b^*	0.007
Phenylalanine	2.48 × 10^−4^ (7.34 × 10^−5^) *^ab^*	3.37 × 10^−4^ (7.77 × 10^−5^) *^a^*	2.39 × 10^−4^ (4.65 × 10^−5^) *^b^*	0.013
Threonine	3.25 × 10^−4^ (1.88 × 10^−4^) *^a^*	4.27 × 10^−4^ (1.55 × 10^−4^) *^a^*	2.49 × 10^−4^ (4.85 × 10^−5^) *^b^*	0.008
Tyrosine	1.86 × 10^−4^ (2.99 × 10^−5^) *^ab^*	2.02 × 10^−4^ (5.03 × 10^−5^) *^a^*	1.60 × 10^−4^ (3.46 × 10^−5^) *^b^*	0.011
Valine	3.28 × 10^−4^ (4.58 × 10^−5^) *^ab^*	3.99 × 10^−4^ (7.97 × 10^−5^) *^a^*	3.03 × 10^−4^ (7.23 × 10^−5^) *^b^*	0.019
Mannose	2.73 × 10^−4^ (4.26 × 10^−5^) *^ab^*	3.11 × 10^−4^ (9.82 × 10^−5^) *^a^*	2.35 × 10^−4^ (3.08 × 10^−5^) *^b^*	0.039
Formate	7.43 × 10^−5^ (7.88 × 10^−6^) *^ab^*	6.94 × 10^−5^ (1.85 × 10^−5^) *^b^*	8.34 × 10^−5^ (5.95 × 10^−6^) *^a^*	0.012
IMP	1.39 × 10^−4^ (1.74 × 10^−4^) *^ab^*	4.30 × 10^−4^ (2.29 × 10^−4^) *^a^*	1.20 × 10^−4^ (3.44 × 10^−5^) *^b^*	0.031
Inosine	1.73 × 10^−4^ (1.35 × 10^−4^) *^ab^*	3.81 × 10^−4^ (1.89 × 10^−4^) *^a^*	1.76 × 10^−4^ (4.33 × 10^−5^) *^b^*	0.021

^1^ Median values followed by different letters are significantly different at *p* < 0.05.

## Supplementary Materials

The following are available online at https://www.mdpi.com/2304-8158/9/4/481/s1, Figures S1: Graphic design of the workflow for meat samples preparation and ^1^H-NMR spectra processing, Figures S2–S13: Above panel - portions of the spectra, superimposed in white-washed mode. The black and red dashed lines show the portions of the spectra employed for the quantification of each molecule. Below – one representative spectrum (black line) superimposed to the signals simulated by software Chenomx (red line) for each of the molecules listed; Table S1: Concentrations (mmol/g, median (IQR)) of molecules quantified by ^1^H-NMR.
