# Supplementary material for: An Untargeted Metabolomics Investigation of Jiulong Yak (Bos grunniens) Meat by 1H-NMR
Source: Foods. 2020 Apr 12;9(4):481. doi: 10.3390/foods9040481 (PMC7230376; doi:10.3390/foods9040481)
Supplement: Supplementary file 1 [file foods-09-00481-s001.pdf]

An untargeted metabolomics investigation of Jiulong Yak (*Bos grunniens*) meat by ^{1}H-NMR

Chenglin Zhu[1], Massimiliano Petracci[1], Cheng Li[2], Enrico Fiore[3] and Luca Laghi[1,*]

SUPPLEMENTAL MATERIAL

Table S1 Concentrations (mmol/g, median (IQR)) of molecules quantified by ^{1}H-NMR

	Triceps brachii (TB)	*Biceps femoris* (BF)	*Longissimus thoracis* (LT)	*p*	*Trapezius* (TP)	Fold change (LT/TP)
**Amino acids, Peptides and analogues**						
Alanine	4.76×10^{-3} (1.33×10^{-3}) ^{a}	5.31×10^{-3} (4.77×10^{-4}) ^{a}	3.14×10^{-3} (9.50×10^{-4}) ^{b}	0.003	3.56×10^{-3} (2.59×10^{-5})	0.88
Anserine	1.27×10^{-3} (5.68×10^{-4}) ^{a}	1.54×10^{-3} (6.66×10^{-4}) ^{a}	1.94×10^{-3} (6.45×10^{-4}) ^{a}	0.164	1.06×10^{-3} (3.62×10^{-6})	1.83
beta-Alanine	1.14×10^{-4} (1.21×10^{-4}) ^{a}	1.50×10^{-4} (1.84×10^{-4}) ^{a}	1.12×10^{-4} (8.98×10^{-5}) ^{a}	0.968	1.26×10^{-4} (1.36×10^{-5})	0.89
Betaine	2.39×10^{-3} (3.90×10^{-4}) ^{a}	2.15×10^{-3} (5.25×10^{-4}) ^{a}	1.79×10^{-3} (1.00×10^{-3}) ^{a}	0.301	2.57×10^{-3} (4.09×10^{-5})	0.70
Carnosine	1.23×10^{-2} (1.65×10^{-3}) ^{b}	1.60×10^{-2} (8.17×10^{-3}) ^{b}	2.09×10^{-2} (1.84×10^{-3}) ^{a}	0.017	1.45×10^{-2} (2.97×10^{-4})	1.44
Creatine	3.89×10^{-2} (2.29×10^{-3}) ^{a}	3.68×10^{-2} (5.63×10^{-3}) ^{a}	4.11×10^{-2} (8.27×10^{-4}) ^{a}	0.389	3.78×10^{-2} (2.20×10^{-4})	1.09
Creatinine	5.71×10^{-4} (7.74×10^{-5}) ^{a}	4.95×10^{-4} (2.89×10^{-4}) ^{a}	7.84×10^{-4} (8.11×10^{-5}) ^{a}	0.094	6.22×10^{-4} (6.66×10^{-5})	1.26
Glutamate	1.06×10^{-3} (2.77×10^{-4}) ^{a}	1.41×10^{-3} (5.55×10^{-4}) ^{a}	7.68×10^{-4} (1.83×10^{-4}) ^{a}	0.096	1.20×10^{-3} (9.25×10^{-5})	0.64
Glutamine	3.75×10^{-3} (3.15×10^{-3}) ^{a}	3.26×10^{-3} (1.28×10^{-3}) ^{a}	1.49×10^{-3} (1.63×10^{-4}) ^{a}	0.051	3.87×10^{-3} (1.35×10^{-4})	0.38*
Glutathione	2.47×10^{-4} (9.01×10^{-6}) ^{a}	2.80×10^{-4} (1.84×10^{-4}) ^{a}	1.50×10^{-4} (5.71×10^{-5}) ^{a}	0.136	1.96×10^{-4} (3.48×10^{-5})	0.77
Glycine	2.26×10^{-3} (1.13×10^{-3}) ^{a}	2.25×10^{-3} (4.31×10^{-4}) ^{a}	2.10×10^{-3} (2.49×10^{-4}) ^{a}	0.280	1.77×10^{-3} (2.11×10^{-5})	1.18
Isoleucine	1.81×10^{-4} (3.39×10^{-5}) ^{ab}	2.33×10^{-4} (6.07×10^{-5}) ^{a}	1.69×10^{-4} (4.61×10^{-5}) ^{b}	0.012	2.27×10^{-4} (1.19×10^{-5})	0.74
Leucine	3.54×10^{-4} (7.22×10^{-5}) ^{ab}	4.70×10^{-4} (9.67×10^{-5}) ^{a}	3.06×10^{-4} (9.42×10^{-5}) ^{b}	0.007	4.31×10^{-4} (1.60×10^{-5})	0.71
Methionine	1.69×10^{-3} (5.95×10^{-4}) ^{a}	1.25×10^{-3} (1.39×10^{-3}) ^{a}	1.68×10^{-3} (1.29×10^{-3}) ^{a}	0.597	5.14×10^{-3} (6.09×10^{-4})	0.33*
N,N-Dimethylglycine	1.13×10^{-4} (6.30×10^{-6}) ^{a}	1.06×10^{-4} (1.06×10^{-5}) ^{a}	1.17×10^{-4} (4.17×10^{-6}) ^{a}	0.479	1.10×10^{-4} (1.54×10^{-6})	1.06
Phenylalanine	2.48×10^{-4} (7.34×10^{-5}) ^{ab}	3.37×10^{-4} (7.77×10^{-5}) ^{a}	2.39×10^{-4} (4.65×10^{-5}) ^{b}	0.013	3.10×10^{-4} (1.43×10^{-5})	0.77
Proline	1.80×10^{-4} (3.42×10^{-5}) ^{a}	1.68×10^{-4} (5.06×10^{-5}) ^{a}	1.35×10^{-4} (4.95×10^{-5}) ^{a}	0.074	2.06×10^{-4} (2.28×10^{-5})	0.66
Taurine	3.05×10^{-3} (3.50×10^{-4}) ^{a}	2.96×10^{-3} (9.75×10^{-3}) ^{a}	2.88×10^{-3} (3.22×10^{-3}) ^{a}	0.178	1.42×10^{-3} (2.03×10^{-5})	2.03*
Threonine	3.25×10^{-4} (1.88×10^{-4}) ^{a}	4.27×10^{-4} (1.55×10^{-4}) ^{a}	2.49×10^{-4} (4.85×10^{-5}) ^{b}	0.008	3.78×10^{-4} (4.45×10^{-5})	0.66
Tyrosine	1.86×10^{-4} (2.99×10^{-5}) ^{ab}	2.02×10^{-4} (5.03×10^{-5}) ^{a}	1.60×10^{-4} (3.46×10^{-5}) ^{b}	0.011	2.37×10^{-4} (1.13×10^{-5})	0.67
Valine	3.28×10^{-4} (4.58×10^{-5}) ^{ab}	3.99×10^{-4} (7.97×10^{-5}) ^{a}	3.03×10^{-4} (7.23×10^{-5}) ^{b}	0.019	3.85×10^{-4} (9.49×10^{-6})	0.79
**Carbohydrates and Derivates**						
1,3-Dihydroxyacetone	2.06×10^{-5} (9.59×10^{-6}) ^{a}	2.08×10^{-5} (3.54×10^{-6}) ^{a}	1.64×10^{-5} (4.54×10^{-6}) ^{a}	0.875	1.97×10^{-5} (2.59×10^{-6})	0.83
Glucose	5.89×10^{-3} (7.36×10^{-4}) ^{a}	6.17×10^{-3} (1.08×10^{-3}) ^{a}	6.06×10^{-3} (5.26×10^{-5}) ^{a}	0.950	6.18×10^{-3} (4.29×10^{-4})	0.89
Glucose-1-phosphate	4.95×10^{-4} (2.01×10^{-4}) ^{a}	4.52×10^{-4} (1.14×10^{-4}) ^{a}	5.68×10^{-4} (1.63×10^{-4}) ^{a}	0.908	2.88×10^{-4} (1.21×10^{-5})	1.97
Mannose	2.73×10^{-4} (4.26×10^{-5}) ^{ab}	3.11×10^{-4} (9.82×10^{-5}) ^{a}	2.35×10^{-4} (3.08×10^{-5}) ^{b}	0.039	1.77×10^{-4} (3.60×10^{-5})	1.33
**Organic acids and Derivates**						
2-Oxovalerate	5.95×10^{-5} (5.19×10^{-5}) ^{a}	7.42×10^{-5} (4.89×10^{-5}) ^{a}	6.78×10^{-5} (4.85×10^{-5}) ^{a}	0.618	8.13×10^{-5} (1.47×10^{-5})	0.83
4-Hydroxybutyrate	8.86×10^{-5} (9.80×10^{-5}) ^{a}	1.09×10^{-4} (8.26×10^{-5}) ^{a}	7.61×10^{-5} (5.59×10^{-5}) ^{a}	0.390	5.96×10^{-5} (8.73×10^{-6})	1.28

Acetate	3.23x10^{-4} (6.11x10^{-5}) ^{a}	5.45x10^{-4} (2.67x10^{-4}) ^{a}	4.11x10^{-4} (2.39x10^{-4}) ^{a}	0.180	6.08x10^{-4} (1.08x10^{-4})	0.68
Acetoacetate	3.51x10^{-5} (5.08x10^{-6}) ^{a}	3.65x10^{-5} (7.37x10^{-6}) ^{a}	1.90x10^{-5} (4.20x10^{-6}) ^{a}	0.108	2.67x10^{-5} (3.90x10^{-6})	0.71
Citrate	2.11x10^{-4} (6.38x10^{-5}) ^{a}	1.29x10^{-4} (9.36x10^{-5}) ^{a}	1.05x10^{-4} (5.27x10^{-5}) ^{a}	0.274	1.16x10^{-4} (1.53x10^{-5})	0.90
Formate	7.43x10^{-5} (7.88x10^{-6}) ^{ab}	6.94x10^{-5} (1.85x10^{-5}) ^{b}	8.34x10^{-5} (5.95x10^{-6}) ^{a}	0.012	1.01x10^{-4} (1.76x10^{-5})	0.82
Fumarate	1.75x10^{-4} (5.45x10^{-5}) ^{a}	1.58x10^{-4} (3.61x10^{-5}) ^{a}	1.07x10^{-4} (2.55x10^{-5}) ^{a}	0.235	1.68x10^{-4} (3.49x10^{-6})	0.64
Lactate	9.91x10^{-2} (1.18x10^{-2}) ^{a}	9.79x10^{-2} (1.60x10^{-2}) ^{a}	1.03x10^{-1} (9.46x10^{-3}) ^{a}	0.464	9.82x10^{-2} (1.04x10^{-3})	1.05
Malate	1.21x10^{-3} (4.08x10^{-4}) ^{a}	1.36x10^{-3} (2.85x10^{-4}) ^{a}	1.06x10^{-3} (1.02x10^{-4}) ^{a}	0.147	1.28x10^{-3} (5.29x10^{-5})	0.83
Malonate	1.18x10^{-3} (6.98x10^{-5}) ^{a}	1.24x10^{-3} (3.85x10^{-4}) ^{a}	8.21x10^{-4} (3.50x10^{-4}) ^{a}	0.258	9.72x10^{-4} (3.80x10^{-5})	0.84
Pyruvate	2.46x10^{-4} (1.71x10^{-4}) ^{a}	1.40x10^{-4} (8.06x10^{-5}) ^{a}	1.99x10^{-4} (1.61x10^{-4}) ^{a}	0.656	1.17x10^{-4} (3.04x10^{-6})	1.70
**Nucleosides, Nucleotides and Analogues**						
Hypoxanthine	5.01x10^{-3} (1.62x10^{-3}) ^{a}	5.55x10^{-3} (1.10x10^{-3}) ^{a}	4.24x10^{-3} (4.07x10^{-4}) ^{a}	0.426	9.29x10^{-3} (1.63x10^{-4})	0.46*
IMP	1.39x10^{-4} (1.74x10^{-4}) ^{ab}	4.30x10^{-4} (2.29x10^{-4}) ^{a}	1.20x10^{-4} (3.44x10^{-5}) ^{b}	0.031	4.18x10^{-4} (3.91x10^{-5})	0.28*
Inosine	1.73x10^{-4} (1.35x10^{-4}) ^{ab}	3.81x10^{-4} (1.89x10^{-4}) ^{a}	1.76x10^{-4} (4.33x10^{-5}) ^{b}	0.021	3.97x10^{-4} (2.68x10^{-5})	0.44*
myo-Inositol	1.46x10^{-3} (3.25x10^{-3}) ^{a}	5.94x10^{-4} (2.56x10^{-3}) ^{a}	1.71x10^{-4} (2.68x10^{-4}) ^{a}	0.176	1.49x10^{-3} (1.02x10^{-4})	0.11*
Niacinamide	4.77x10^{-4} (1.06x10^{-4}) ^{a}	5.34x10^{-4} (1.20x10^{-4}) ^{a}	5.79x10^{-4} (4.80x10^{-5}) ^{a}	0.695	5.14x10^{-4} (6.78x10^{-6})	1.13
UMP	6.91x10^{-5} (1.81x10^{-5}) ^{a}	7.74x10^{-5} (2.58x10^{-5}) ^{a}	8.89x10^{-5} (2.03x10^{-5}) ^{a}	0.511	4.89x10^{-5} (1.77x10^{-6})	1.82
Uridine	8.95x10^{-5} (2.35x10^{-5}) ^{a}	8.84x10^{-5} (2.11x10^{-5}) ^{a}	9.04x10^{-5} (7.09x10^{-6}) ^{a}	0.794	1.36x10^{-4} (1.30x10^{-5})	0.66
Xanthine	2.82x10^{-4} (6.33x10^{-5}) ^{a}	2.55x10^{-4} (4.63x10^{-5}) ^{a}	3.09x10^{-4} (2.66x10^{-5}) ^{a}	0.240	2.87x10^{-4} (1.60x10^{-5})	1.08
**Miscellaneous**						
Acetoin	1.16x10^{-5} (1.50x10^{-6}) ^{a}	1.12x10^{-5} (3.68x10^{-5}) ^{a}	1.52x10^{-5} (6.62x10^{-5}) ^{a}	0.428	4.46x10^{-6} (1.63x10^{-6})	3.42*
Carnitine	8.77x10^{-3} (2.79x10^{-3}) ^{a}	6.36x10^{-3} (8.25x10^{-4}) ^{a}	5.30x10^{-3} (1.41x10^{-3}) ^{a}	0.127	7.21x10^{-3} (6.45x10^{-4})	0.73
Choline	9.71x10^{-4} (6.22x10^{-4}) ^{a}	5.44x10^{-4} (7.76x10^{-4}) ^{a}	1.02x10^{-3} (7.20x10^{-4}) ^{a}	0.727	3.44x10^{-3} (4.36x10^{-4})	0.30*
Dimethyl sulfone	1.08x10^{-4} (5.30x10^{-6}) ^{a}	9.97x10^{-5} (1.60x10^{-5}) ^{a}	1.15x10^{-4} (6.61x10^{-6}) ^{a}	0.597	1.03x10^{-4} (1.59x10^{-6})	1.12
Guanidoacetate	1.79x10^{-3} (7.67x10^{-4}) ^{a}	2.27x10^{-3} (8.18x10^{-4}) ^{a}	2.53x10^{-3} (5.43x10^{-4}) ^{a}	0.107	1.40x10^{-3} (2.68x10^{-5})	1.80
Glycerol	4.96x10^{-3} (1.40x10^{-3}) ^{a}	6.39x10^{-3} (1.32x10^{-3}) ^{a}	5.63x10^{-3} (1.36x10^{-3}) ^{a}	0.101	3.97x10^{-3} (1.62x10^{-4})	1.42
Methanol	3.25x10^{-4} (6.03x10^{-4}) ^{a}	1.42x10^{-4} (3.73x10^{-4}) ^{a}	7.63x10^{-5} (3.93x10^{-5}) ^{a}	0.114	1.71x10^{-4} (5.24x10^{-6})	0.45*
O-Acetylcarnitine	6.25x10^{-4} (3.56x10^{-4}) ^{a}	3.60x10^{-4} (4.78x10^{-4}) ^{a}	7.23x10^{-4} (3.97x10^{-4}) ^{a}	0.841	2.18x10^{-3} (2.89x10^{-4})	0.33*
Trimethylamine	2.32x10^{-5} (2.38x10^{-6}) ^{a}	2.03x10^{-5} (4.49x10^{-6}) ^{a}	1.49x10^{-5} (4.71x10^{-6}) ^{a}	0.190	2.13x10^{-5} (8.27x10^{-7})	0.70

[1] Means with different letters (a, b and ab) are significantly different at $p<0.05$. * Molecules characterized by a the fold change below 0.5 or above 2 [32].

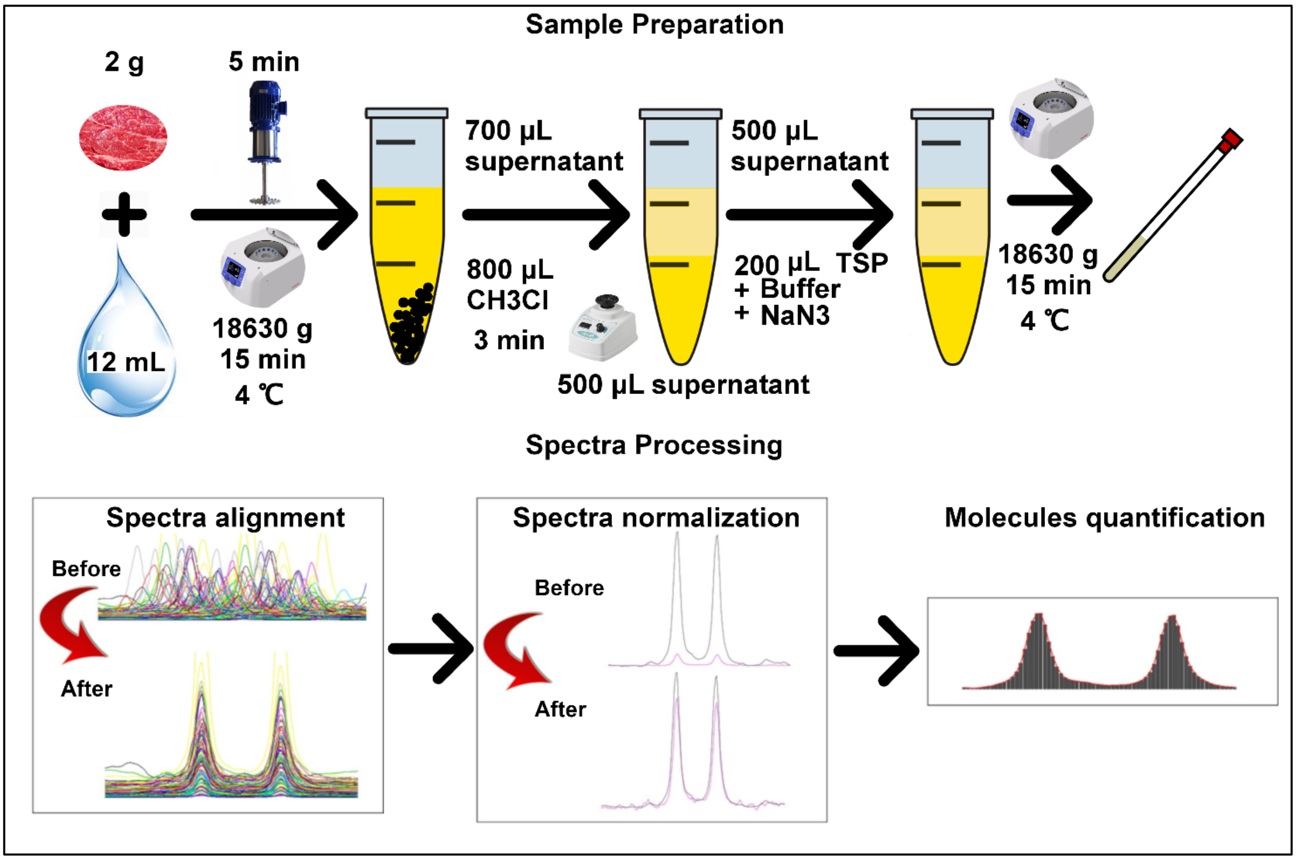

Figure S1. Graphic design of the workflow for meat samples preparation and ^{1}H-NMR spectra processing.

Figures S2-S13: Above panel - portions of the spectra, superimposed in white-washed mode. The black and red dashed lines show the portions of the spectra employed for the quantification of each molecule. Below – one representative spectrum (black line) superimposed to the signals simulated by software Chenomx (red line) for each of the molecules listed.

2

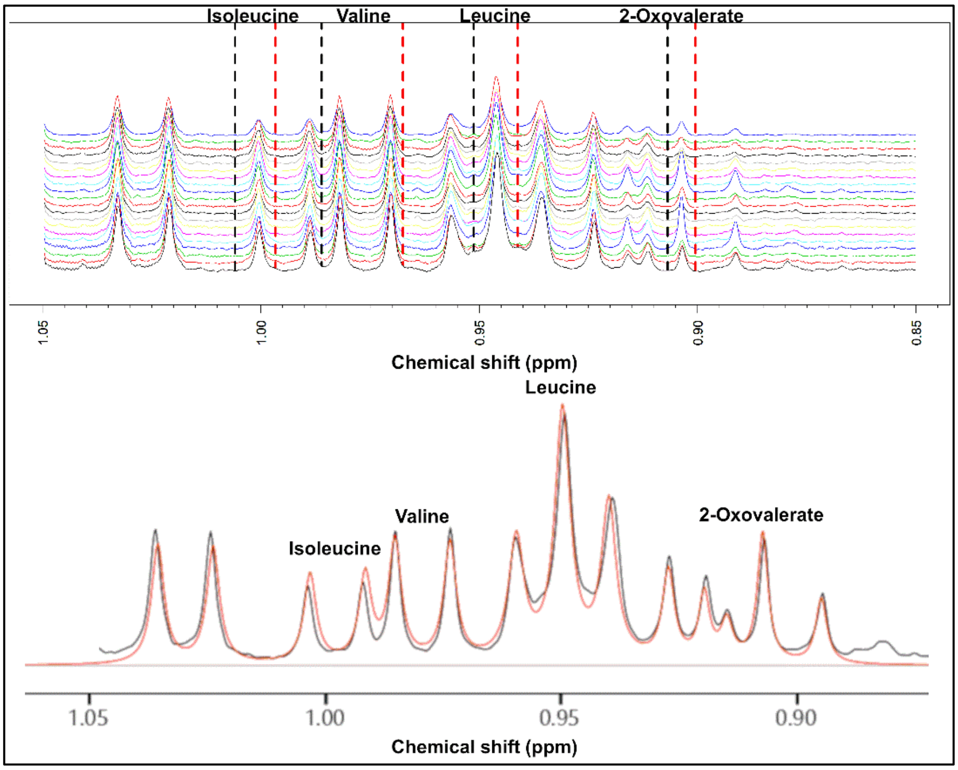

3

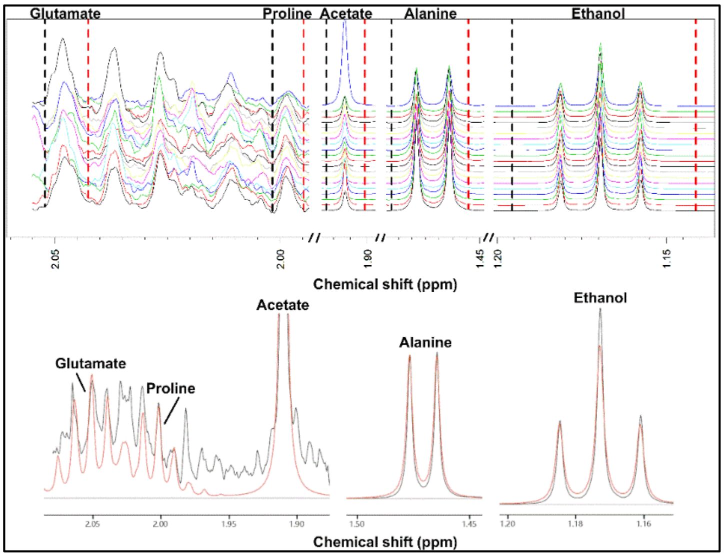

4

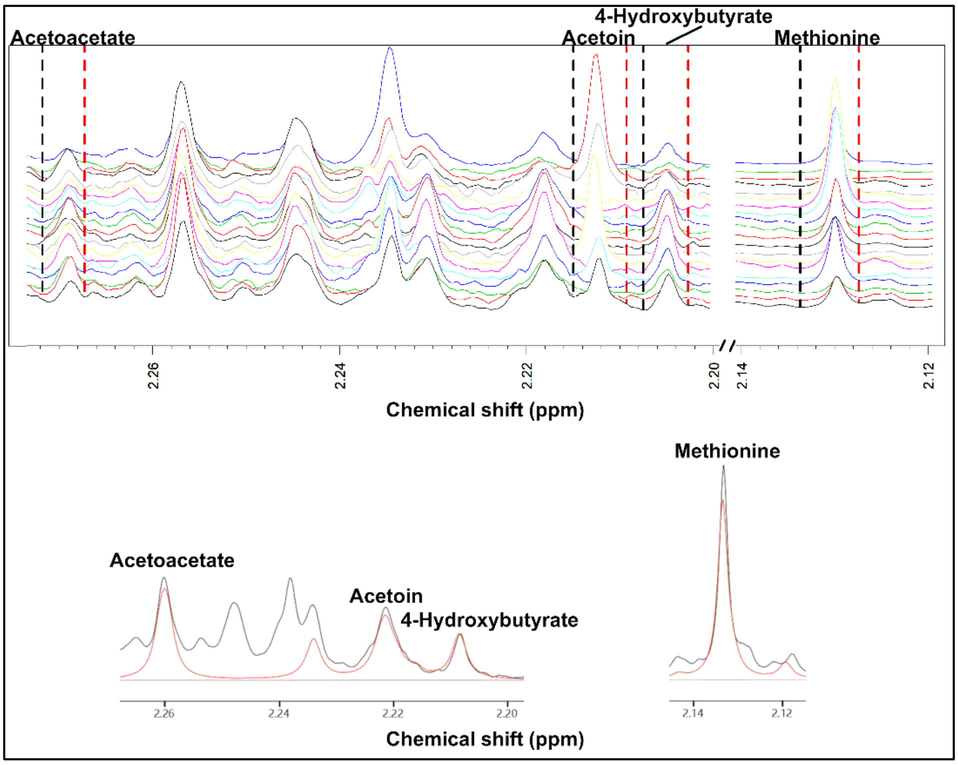
Acetoacetate
4-Hydroxybutyrate
Acetoin
Methionine
2.26
2.24
2.22
2.20
2.14
2.12
Chemical shift (ppm)
Methionine
Acetoacetate
Acetoin
4-Hydroxybutyrate
2.26
2.24
2.22
2.20
2.14
2.12
Chemical shift (ppm)

5

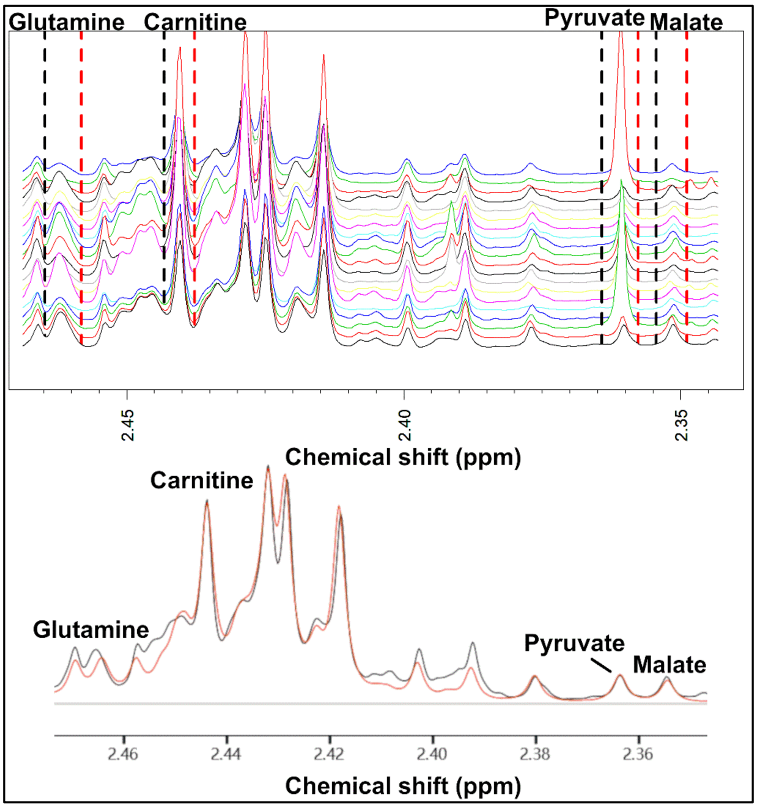
Glutamine
Carnitine
Pyruvate
Malate
2.45
2.40
2.35
Chemical shift (ppm)
Carnitine
Glutamine
Pyruvate
Malate
2.46
2.44
2.42
2.40
2.38
2.36
Chemical shift (ppm)

6

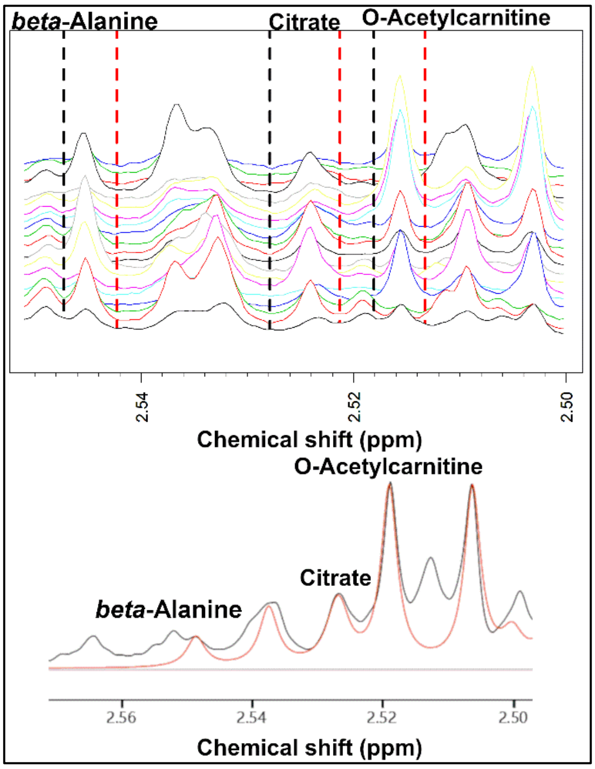

beta-Alanine
Citrate
O-Acetylcarnitine
2.54
2.52
2.50
Chemical shift (ppm)
O-Acetylcarnitine
Citrate
beta-Alanine
2.56
2.54
2.52
2.50
Chemical shift (ppm)

7

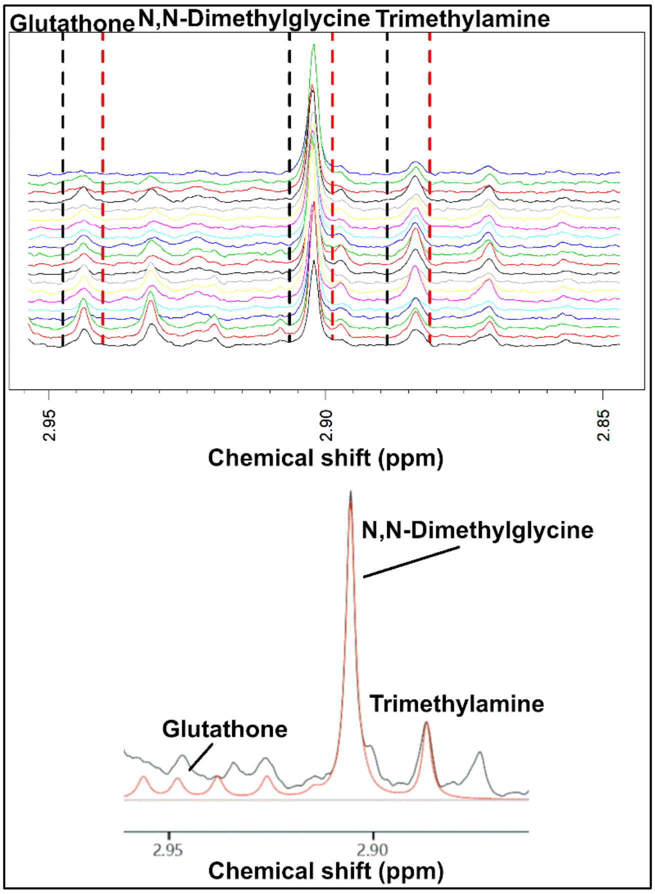

Glutathone
N,N-Dimethylglycine
Trimethylamine
2.95
2.90
2.85
Chemical shift (ppm)
N,N-Dimethylglycine
Trimethylamine
Glutathone
2.95
2.90
Chemical shift (ppm)

8

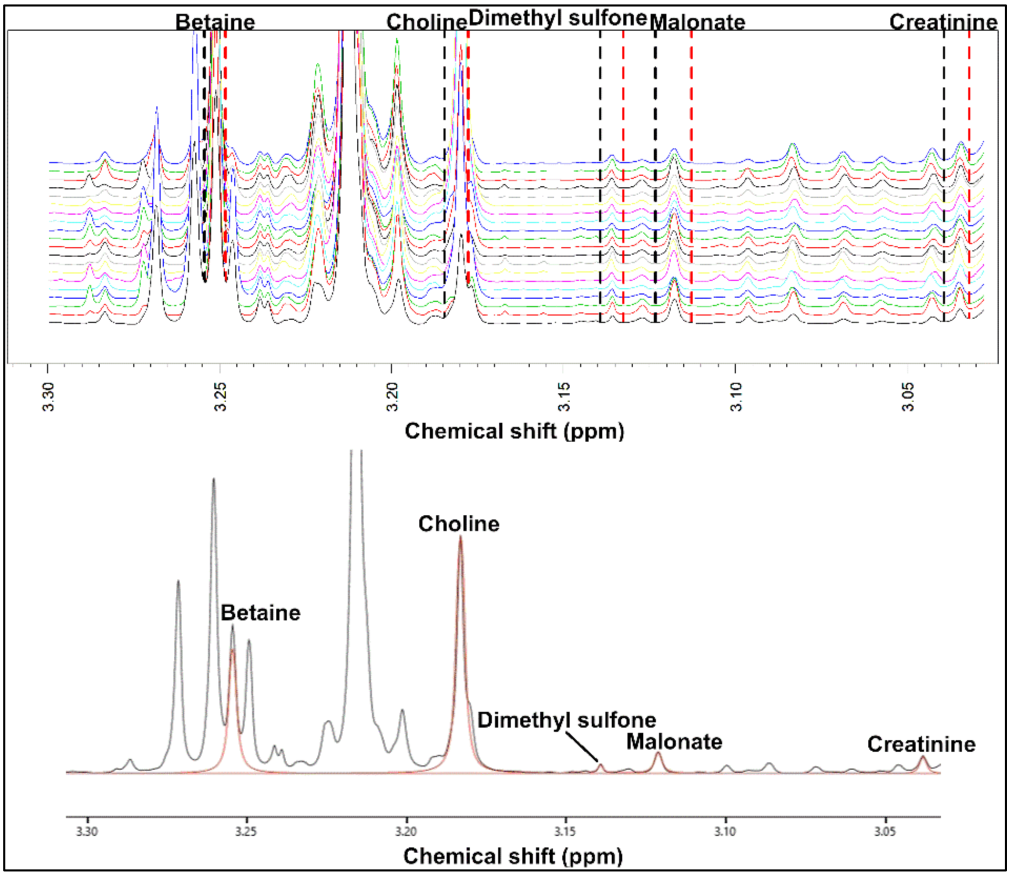
Betaine
Choline
Dimethyl sulfone
Malonate
Creatinine
3.30
3.25
3.20
3.15
3.10
3.05
Chemical shift (ppm)
Choline
Betaine
Dimethyl sulfone
Malonate
Creatinine
3.30
3.25
3.20
3.15
3.10
3.05
Chemical shift (ppm)

9

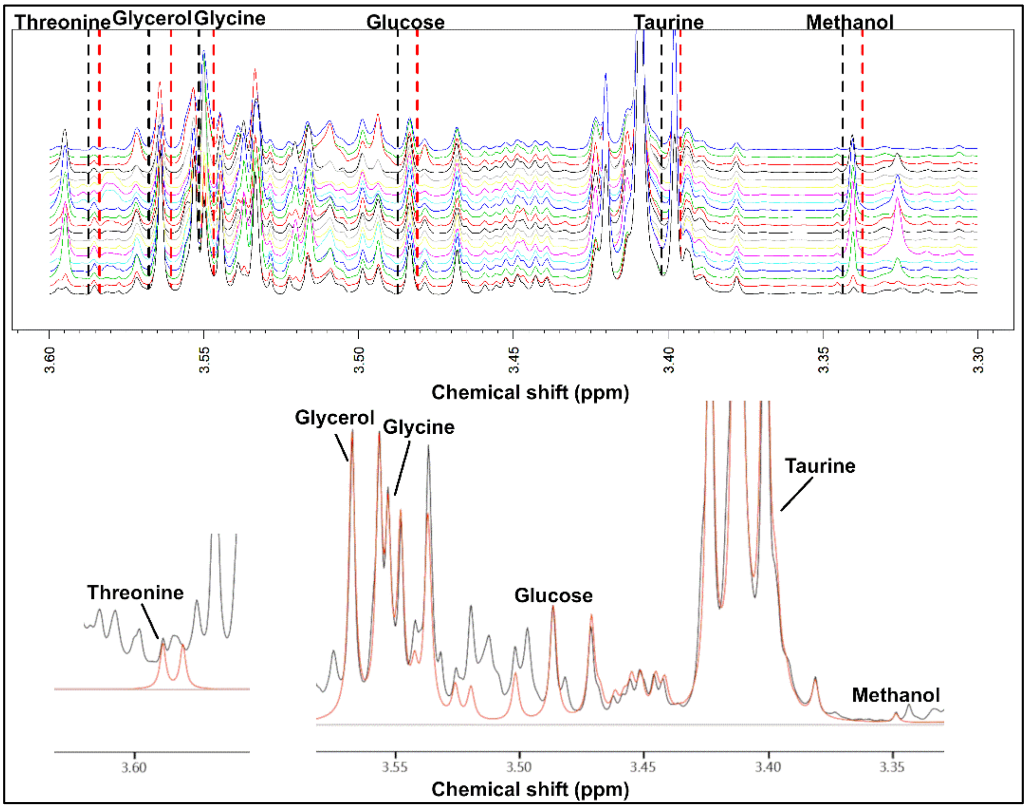
Threonine
Glycerol
Glycine
Glucose
Taurine
Methanol
3.60
3.55
3.50
3.45
3.40
3.35
3.30
Chemical shift (ppm)
Glycerol
Glycine
Taurine
Threonine
Glucose
Methanol
3.60
3.55
3.50
3.45
3.40
3.35
Chemical shift (ppm)

10

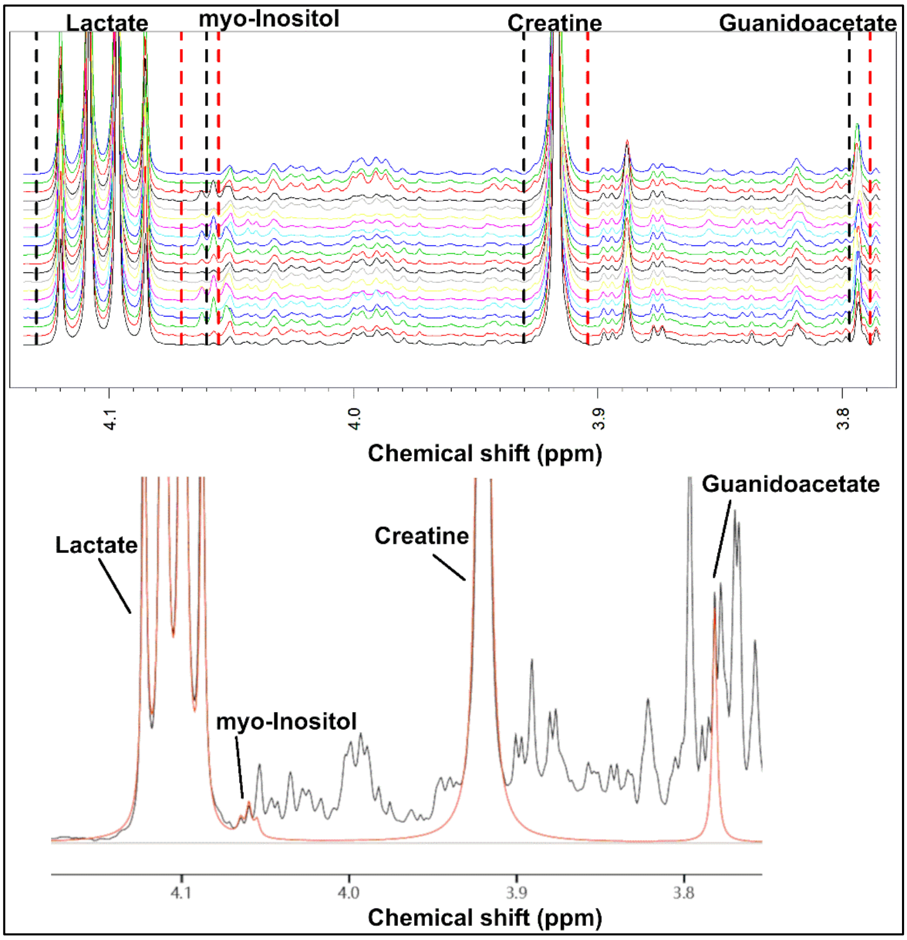
Lactate
myo-Inositol
Creatine
Guanidoacetate
4.1
4.0
3.9
3.8
Chemical shift (ppm)
Guanidoacetate
Creatine
Lactate
myo-Inositol
4.1
4.0
3.9
3.8
Chemical shift (ppm)

11

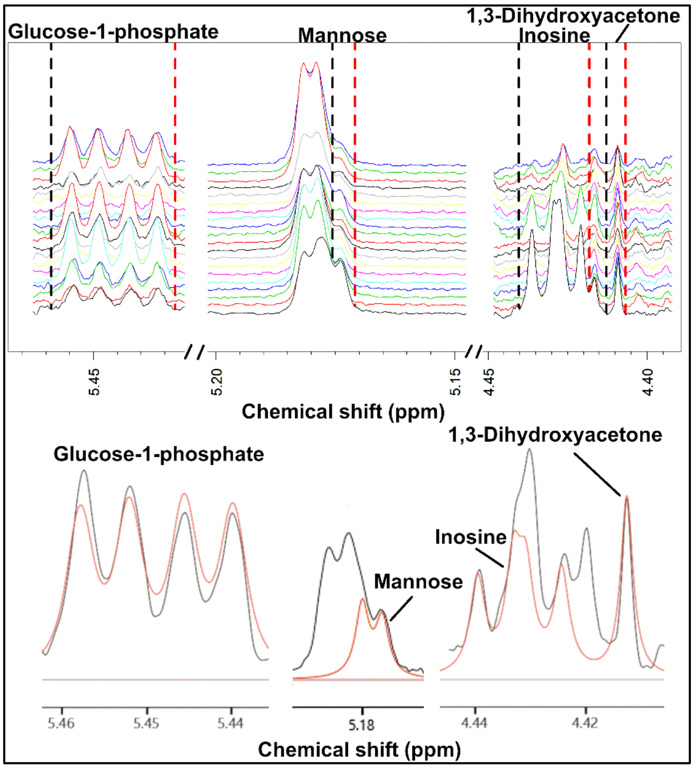
Glucose-1-phosphate
Mannose
1,3-Dihydroxyacetone
Inosine
5.45
5.20
5.15
4.45
4.40
Chemical shift (ppm)
Glucose-1-phosphate
1,3-Dihydroxyacetone
Inosine
Mannose
5.46
5.45
5.44
5.18
4.44
4.42
Chemical shift (ppm)

12

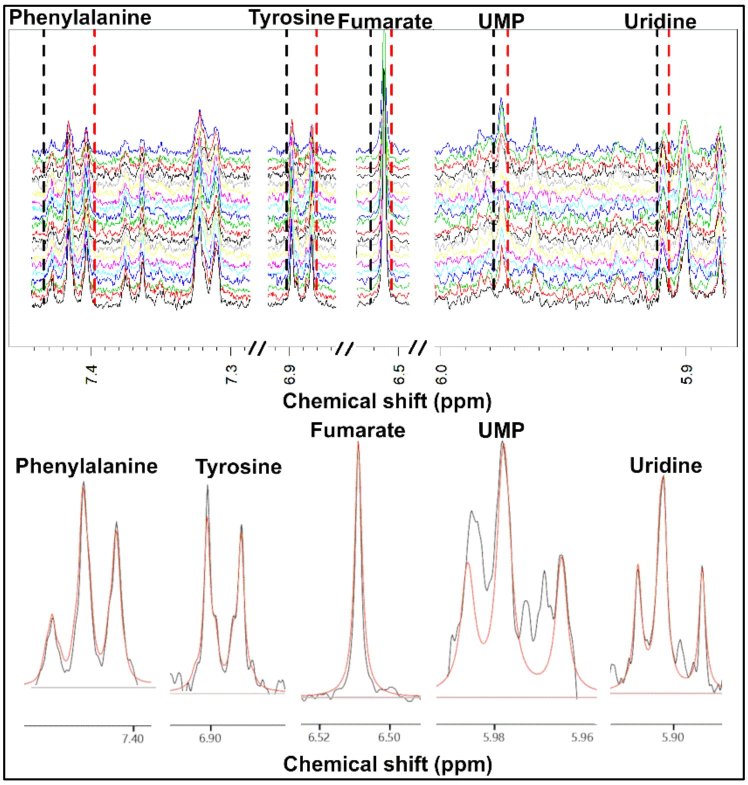
Phenylalanine
Tyrosine
Fumarate
UMP
Uridine
7.4
7.3
6.9
6.5
6.0
5.9
Chemical shift (ppm)
Fumarate
UMP
Phenylalanine
Tyrosine
Uridine
7.40
6.90
6.52
6.50
5.98
5.96
5.90
Chemical shift (ppm)

13

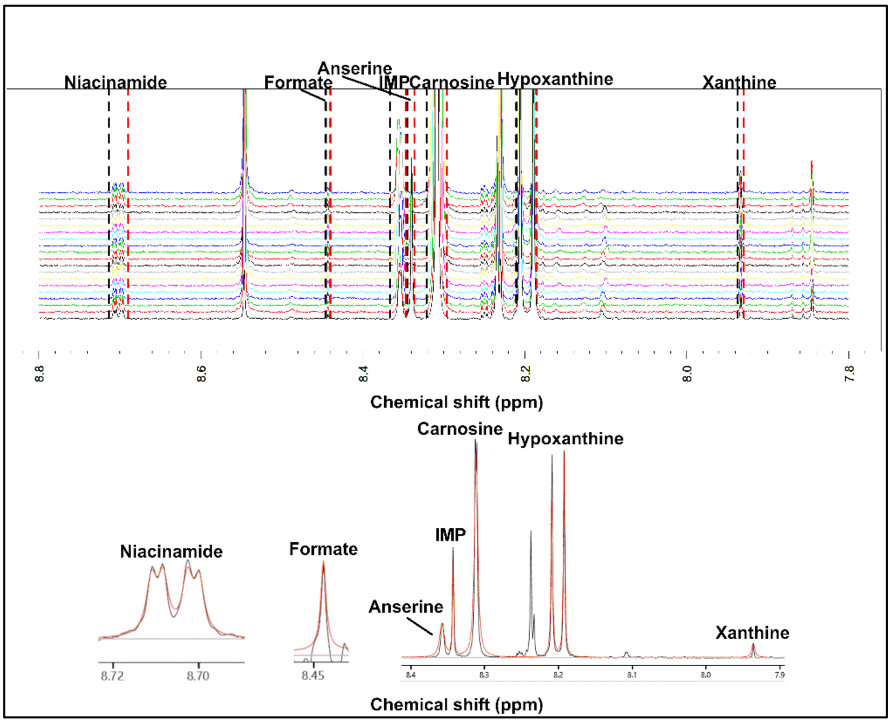
Niacinamide
Formate
Anserine
IMP
Carnosine
Hypoxanthine
Xanthine
8.8
8.6
8.4
8.2
8.0
7.8
Chemical shift (ppm)
Carnosine
Hypoxanthine
IMP
Niacinamide
Formate
Anserine
Xanthine
8.72
8.70
8.45
Chemical shift (ppm)
